# Experimental Study on Static Strength and Fatigue of CNT-Modified CFRP Laminates

**DOI:** 10.3390/polym18192368

**Published:** 2026-09-28

**Authors:** Yusong Qi, Junhao Zhang, Pinqi Xia

**Affiliations:** Institute of Helicopter Engineering, Nanjing University of Aeronautics and Astronautics, Nanjing 210016, China; nuaaqys@nuaa.edu.cn

**Keywords:** carbon nanotubes, composites, static strength, fatigue life, experiments

## Abstract

In this paper, an experimental study on the static strength and fatigue of carbon fiber-reinforced polymer (CFRP) laminates with ply angles of 0°, ±15° and ±45° and single-walled carbon nanotubes (SWCNTs) of 3% mass fraction in epoxy matrix has been presented to systematically demonstrate the SWCNT-modified effect on the static tensile properties and fatigue behavior of the CFRP laminates. The results of pseudo-static tensile tests show that the addition of SWCNTs increases the tensile strength of the three laminates by 37%, 43%, and 34%, respectively, and the elastic modulus by 5%, 62%, and 35%, respectively. The S-N curves obtained by the fatigue tests at various stress levels under graded tension–tension cyclic loads demonstrate that the SWCNT-modified laminates sustain higher stress levels than those without SWCNTs at the same fatigue cycles, and with the most obvious improvement of ±15° laminates. The fracture surface observations of the laminates indicate that the enhanced performance is attributed to the synergistic effects of matrix modification, interfacial bonding improvement and crack bridging by SWCNTs. Therefore, moderate addition of SWCNTs in the epoxy matrix of CFRP laminates is an effective approach for improving both the static strength and fatigue performance of the laminates.

## 1. Introduction

Carbon nanotubes (CNTs) have been recognized as ideal nanofillers for reinforcing polymer matrix composites, owing to their ultra-high aspect ratio, exceptional mechanical properties, electrical and thermal conductivities, and unique interfacial tailoring capabilities [1]. Numerous studies have shown that introducing CNTs into carbon fiber-reinforced polymer (CFRP) systems could effectively inhibit microcrack initiation and propagation through mechanisms such as crack bridging, fiber pull-out, and crack deflection, thereby enhancing the interlaminar shear strength, fracture toughness, and fatigue performance of composites [2]. Currently, modification strategies for CFRP using CNTs are primarily categorized into two fundamental approaches: fiber modification and matrix modification. Fiber modification includes methods such as surface grafting, in situ synthesis, and interlaminar insertion, all of which introduce CNTs onto the fiber surfaces or into the interlaminar regions to improve interfacial bonding and stress transfer. Matrix modification involves uniformly dispersing CNTs in the epoxy resin or other matrices to directly enhance the matrix strength, modulus, and toughness. Among these, matrix modification has become the most practically valuable pathway due to its simple processing, strong compatibility, and ease of industrial scale-up [3]. Matrix modification directly enhances matrix strength, modulus, and toughness by uniformly dispersing CNTs in epoxy resin or other matrices, while simultaneously optimizing the interfacial bonding and stress transfer between fibers and the matrix, thereby achieving synergistic improvement in overall composite performance [4].

Mirsalehi et al. employed a filament winding process, achieving uniform CNT dispersion in the resin through ultrasonication and mechanical stirring, significantly enhancing the transverse static strength and interfacial properties of CFRP [5]. Li et al. systematically reviewed research progress on CNT-modified FRP composites, summarizing four mainstream methods—matrix dispersion, in situ fiber growth, prepreg modification, and CNT film insertion—and confirmed that CNTs can enhance interlaminar toughness, impact resistance, and fatigue life through crack bridging and pull-out effects [6]. Ismail et al. reviewed research findings on the fatigue fracture performance of CNT-toughened polymer matrix composites, establishing that CNTs can enhance fracture toughness and fatigue life through energy dissipation mechanisms, while noting that existing research still has deficiencies in multi-factor coupling and macroscopic life prediction, which underscores the need for the present fatigue life evaluation [7]. Boroujeni and Al-Haik employed the graphitic structures by design (GSD) low-temperature synthesis technology to controllably grow patterned CNTs on carbon fiber fabric surfaces, significantly enhancing interfacial bonding and suppressing matrix crack propagation, thereby simultaneously improving the static strength and fatigue life of CFRP, providing a basis for the comparative analysis of static and fatigue properties in this study [8]. Davis et al. used amine-functionalized CNTs for CFRP interfacial reinforcement through solvent spraying and heat-assisted molding, effectively enhancing the static strength, stiffness, and tension–tension fatigue life while suppressing interfacial cracking and delamination failure, indicating that interface optimization is an effective strategy for improving overall performance [9]. Matveeva et al. employed dual-scale finite element and cohesive zone models to theoretically confirm that CNTs on fiber surfaces can reduce interfacial stress concentration and slow debonding propagation, providing theoretical support for understanding the reinforcing mechanisms of CNTs [10]. Jen et al. demonstrated that adding carbon nanoparticles to the matrix can significantly improve the impact resistance and fatigue damage tolerance of carbon fiber/epoxy composites, effectively suppressing fatigue damage propagation, which further supports the choice of the matrix modification route in this study [11]. Barkauskas et al. confirmed through fatigue tests that CNTs can stabilize the initial stiffness of composites and mitigate internal cumulative damage under variable amplitude loading, providing a reference for the stiffness degradation analysis in this work [12]. Soni et al. noted in their review that CNTs can effectively improve interfacial bonding, enhance static strength and toughness, and provide a reliable pathway for extending composite fatigue life, which is consistent with the mechanistic interpretation in this paper [13]. Li et al. demonstrated that CNT film interlaminar toughening can suppress crack propagation and enhance the interlaminar toughness and mechanical properties of the composites, which contrasts with the matrix modification approach adopted in this study [14]. Shen et al. demonstrated that incorporating CNT buckypaper as an interleaf can significantly decrease interlaminar stresses and enhance the ultimate strength of composite laminates, further illustrating the reinforcing potential of CNTs in composite materials [15].

Recent advances in carbonaceous nanomaterial-reinforced polymer nanocomposites provide a broader context for this study. Latif et al. systematically reviewed the thermomechanical properties of nano-carbon-reinforced polymeric nanocomposites, covering various carbon nanomaterials including graphene, carbon nanotubes, carbon black, and fullerene [16]. Navidfar and Trabzon further highlighted the synergistic effects of hybrid CNT/graphene fillers in polymer nanocomposites, demonstrating that the combination of different dimensional carbon nanofillers can achieve greater performance improvement [17]. In the aerospace sector, Kausar reviewed the application of carbonaceous nanofiller-reinforced composites, emphasizing that their performance is highly dependent on nanofiller dispersion, alignment, interfacial interactions, and processing techniques [18]. Kausar and Ahmad reviewed the electrospinning processing of polymer/nanocarbon nanocomposite nanofibers [19], and Subramanian and Bisagni investigated CNT interleaves for toughening composite interfaces [20]. These recent advances underscore the potential of carbonaceous nanomaterials for high-performance composite structures and provide a broader context for the present study on CNT-modified CFRP laminates.

Despite significant progress in CNT-modified CFRP, existing research still has notable limitations: most studies have concentrated on static mechanical property enhancement, while systematic experiments and quantitative analyses on fatigue performance evolution under cyclic loading, S–N curve behavior, and dynamic damage propagation mechanisms are relatively scarce; the structure–property relationship among CNT dispersion uniformity, interfacial bonding strength, and fatigue life remains unclear; and fatigue life prediction models and engineering application data for CNT-reinforced CFRP based on matrix modification at different stress levels still require further development. To address these research gaps, this study incorporates single-walled carbon nanotubes into the epoxy matrix via matrix modification and systematically evaluates their effects on the pseudo-static tensile properties and fatigue behavior of CFRP laminates with three ply angles of 0°, ±15°, and ±45°. The novelty of this work lies in the quantitative assessment of 3 wt% SWCNT on the static strength and fatigue life of CFRP laminates across three ply orientations through S-N curve comparison, and the establishment of correlations between microstructure and macroscopic mechanical properties through microstructural characterization combined with statistical analysis. Accordingly, the following experimental work was carried out. In this paper, we employed single-walled carbon nanotubes (SWCNTs) as the nano-reinforcement phase, prepared SWCNT/epoxy resin impregnation solution via matrix modification, and fabricated CFRP laminate specimens with 0°, ±15°, and ±45° ply orientations through prepreg preparation and layup hot-press molding. Pseudo-static tensile tests and multi-level stress tension–tension fatigue tests of the specimens showed remarkable improvements induced by SWCNTs in the tensile strength, elastic modulus, and fatigue life of CFRP laminates.

## 2. Preparation of CNT-Reinforced Composite Specimens

### 2.1. Materials and Specimen Dimensions

The matrix used in the experiments was E-51 bisphenol A epoxy resin, with 2-ethyl-4-methylimidazole as the curing agent. SWCNTs were employed as the reinforcement phase, and TZ300-3K unidirectional carbon fiber fabric served as the fiber reinforcement. Acetone and vacuum silicone grease release agent were used as auxiliary materials.

The SWCNTs used in this study are amino-functionalized single-walled carbon nanotubes, purchased from OCSiAl (Leudelange, Luxembourg) and Chengdu Organic Chemicals Co., Ltd., Chinese Academy of Sciences (Chengdu, China). The detailed characteristics are as follows: purity over 90%, diameter 1–2 nm, and length 5–30 μm.

To meet the standards for pseudo-static tensile and tension–tension fatigue tests, rectangular laminates with three symmetric layup configurations of 0°, ±15°, and ±45° were prepared, each consisting of 12 symmetrically stacked unidirectional plies with a single-ply thickness of 0.175 mm and a total cured thickness of 2.1 mm. The specimen dimensions were 230 mm in length and 38 mm in width, satisfying gripping and loading space requirements and ensuring uniform stress transfer.

Pseudo-static tensile tests were conducted in accordance with ASTM D3039/D3039M-17 [21]. Aluminum tabs of 50 mm in length and 1.4 mm in thickness were bonded to both ends of each specimen to prevent grip-induced damage and ensure proper load introduction. The gauge length was 50 mm, as measured by the MTS 634.31F-24 multi-gauge-length axial extensometer (MTS Systems Corporation, Eden Prairie, MN, USA).

### 2.2. Preparation of SWCNT/Epoxy Resin Composite Impregnation Solution

An SWCNT mass fraction of 3 wt% was selected based on preliminary tests with 2 wt%, 3 wt%, 4 wt%, and 5 wt% loadings, among which 3 wt% showed the best overall performance in both tensile strength and elastic modulus. To realize homogeneous dispersion of SWCNTs in the epoxy matrix and prevent nanotube agglomeration, a hybrid process integrating ultrasonic treatment with constant-temperature mechanical stirring was employed to fabricate SWCNT/epoxy impregnation solution in this work. SWCNTs were weighed at a mass fraction of 3% (3 wt%) and thoroughly mixed with acetone. The mixture was placed in an ice-water bath and subjected to ultrasonic oscillation using an ultrasonic processor (Model 98-IIIDN, Ningbo Scientz Biotechnology Co., Ltd., Ningbo, China) for 30 min to control the system temperature and enhance the dispersion stability of SWCNTs. SWCNT/acetone dispersion after ultrasonic treatment was slowly added into E-51 epoxy resin, and the mixture was transferred to a constant-temperature oil bath at 60 °C and stirred mechanically at a high speed of 500 r/min for 4.5 h to thoroughly incorporate the SWCNTs into the resin. Subsequently, 5 wt% of 2-ethyl-4-methylimidazole curing agent was added, and stirring was continued for another 0.5 h to ensure uniform dispersion. Finally, a SWCNT/epoxy resin composite impregnation solution with well-dispersed SWCNTs and no obvious agglomeration was obtained.

The dispersion state of 3 wt% SWCNTs in the epoxy matrix was examined by SEM observation (Model S-4700, Hitachi, Tokyo, Japan) of the fracture surface of the SWCNT/epoxy casting, as shown in Figure 1. The SEM image reveals that the SWCNTs are uniformly dispersed in the epoxy matrix without obvious agglomeration, confirming the effectiveness of the adopted dispersion process.

### 2.3. Laminate Compression Molding and Curing Process

Unidirectional carbon fiber fabric was cut to mold dimensions, laid up layer by layer with resin coating, and compacted along the fiber direction after each layer to remove trapped air. The layup was closed and transferred to a flat vulcanizing press (Model CL/GL-01, Shandong Guangxian New Materials Co., Ltd., Jinan, China) for curing following a stepwise heating schedule: 80 °C for 20 min with 2 MPa pressure, 120 °C for 2 h, and 150 °C for 1 h, followed by cooling to room temperature and pressure release. After demolding, the laminates were cut into standard specimens using a computer numerical control machine (Model SC1311, Jingwei Digital Control Equipment Co., Ltd., Jinan, China) and specimens with visible surface defects were discarded. The finished standard specimens are shown in Figure 2.

## 3. Tensile Tests of CNT-Reinforced Composite Specimens

### 3.1. Tensile Test Method

Uniaxial pseudo-static tensile tests were conducted on an MTS Landmark 370.10 electro-hydraulic servo fatigue testing machine (MTS Systems Corporation, Eden Prairie, MN, USA) at room temperature and atmospheric pressure. The experimental setup is shown in Figure 3. During the tests, an MTS 634.31F-24 high-precision multi-gauge-length axial extensometer (MTS Systems Corporation, Eden Prairie, MN, USA) was used to record strain data from the gauge section of the specimens in real time, and the loading rate was set at 2 mm/min. Uniaxial pseudo-static tensile tests were conducted on specimens with and without CNTs for three ply orientations: 0°, ±15°, and ±45°. At least four valid specimens were tested for each condition. Based on the failure load and stress–strain curves obtained from the tests, the tensile strength and elastic modulus of the specimens were calculated.

### 3.2. Analysis of Stress–Strain Curve Characteristics from Tensile Tests

The stress–strain curves of specimens with and without CNTs at ply orientations of 0°, ±15°, and ±45° are shown in Figure 4, Figure 5 and Figure 6, respectively. All specimens exhibited predominantly linear elastic deformation prior to fracture without obvious yield plateaus, demonstrating the typical brittle fracture behavior of carbon fiber/epoxy resin composites.

Figure 4 compares the stress–strain curves of 0° ply specimens with and without CNTs. The unmodified specimens exhibited good linearity with only slight stiffness degradation at high stress levels, while CNT addition further improved the linearity and load-bearing stability with enhanced consistency in fracture strain. Figure 5 presents the results for the ±15° ply, where the unmodified specimens maintained good linearity with minor stiffness degradation at high stress levels, and CNT addition effectively reduced the stiffness degradation. Figure 6 shows the curves for the ±45° ply, where the unmodified specimens displayed pronounced nonlinear bending with noticeable fluctuations, whereas CNT addition resulted in more stable and continuous deformation without obvious fluctuations or hysteresis loops. These results demonstrate that CNTs can stably optimize load transfer, suppress early-stage damage, and improve the mechanical response stability of composites across different ply orientations.

### 3.3. Analysis of Tensile Test Data

The tensile properties of specimens with and without CNTs at different ply orientations are compared in Table 1, with the corresponding improvements and statistical significance summarized in Table 2. The ply orientation had a significant effect on the static mechanical properties of the composites. As the ply orientation increased from 0° to ±45°, the specimens transitioned from axial fiber-dominated loading to shear-dominated loading, with both tensile strength and elastic modulus exhibiting a pronounced decreasing trend.

The 0° ply specimens were dominated by axial loading along the carbon fibers, with the fibers carrying the majority of the load. Consequently, their tensile strength and elastic modulus were significantly higher than those of the other ply orientations, exhibiting the highest static mechanical properties. After the addition of CNTs, the tensile strength of the 0° ply specimens increased from 1104.7 MPa to 1515.5 MPa, and the elastic modulus increased from 121.0 GPa to 127.0 GPa, corresponding to improvements of 37% and 5%, respectively.

The ±15° ply specimens were still dominated by axial fiber loading, accompanied by a small shear component. Their tensile strength and elastic modulus were slightly lower than those of the 0° ply specimens, but overall remained at a relatively high level. After the addition of CNTs, the tensile strength of the ±15° ply specimens increased from 585.5 MPa to 838.0 MPa, and the elastic modulus increased from 73.3 GPa to 118.8 GPa, demonstrating significant improvements, representing improvements of 43% and 62%, respectively.

The ±45° ply specimens were dominated by in-plane shear deformation, with the resin matrix and fiber-matrix interface bearing the main load. Consequently, their tensile strength and elastic modulus were the lowest among the three ply orientations. After the addition of CNTs, the tensile strength of the ±45° ply specimens increased from 144.3 MPa to 193.3 MPa, and the elastic modulus increased from 14.1 GPa to 19.1 GPa, corresponding to improvements of 34% and 35%, respectively.

Statistical significance of the improvements was evaluated using two-tailed Student‘s *t*-tests, with the corresponding *p* and 95% confidence intervals for the percentage improvements presented in Table 2. The results show that CNT addition led to significant improvements in both tensile strength and elastic modulus across all three ply orientations, with *p* < 0.05 for the 0° and ±45° plies and *p* < 0.01 for the ±15° ply. The 95% confidence interval for the ±45° tensile strength improvement is relatively wide, with its lower bound slightly below 0, reflecting the inherent scatter of the experimental data; however, the *p* remains below 0.05, confirming statistical significance. These results confirm that the observed enhancements are statistically significant and not attributable to experimental scatter.

It should be noted that the fiber volume fraction remained essentially unchanged after CNT incorporation. All laminates were fabricated with the same number of plies of 12 layers, the same mold dimensions, and the same curing process, ensuring that both the fiber volume and the total composite volume remained constant across all specimens. The addition of 3 wt% CNTs primarily replaces a small portion of the epoxy matrix rather than altering the fiber content. Therefore, the observed improvements in tensile strength and elastic modulus are attributed to the CNT-induced enhancement of matrix properties and fiber-matrix interfacial bonding, rather than to changes in fiber volume fraction.

Fracture surface morphology of the tensile specimens was examined by SEM. As shown in Figure 7, Figure 8 and Figure 9, CNT pull-out and bridging features are observed on the fracture surfaces of all three ply orientations, including 0°, ±15°, and ±45°. Extensive CNTs are pulled out from the epoxy matrix, with some CNTs embedded at one end in the matrix and exposed at the other end in the fracture voids, exhibiting bending and curling rather than flat fracture. These microstructural features indicate that CNTs effectively transferred load across the crack surfaces and bridged the crack faces during tensile loading, thereby enhancing the resistance to crack propagation. The improved interfacial bonding between CNTs and the epoxy matrix, as evidenced by the pull-out morphology rather than direct debonding, contributes to the enhanced load transfer efficiency from the matrix to the reinforcement. This microstructural mechanism, together with the matrix stiffening effect of CNTs, provides a structural basis for the observed improvements in tensile strength and elastic modulus reported in Table 1. The above experimental data indicate that CNTs improve the tensile strength and elastic modulus of the composites with 0°, ±15°, and ±45° ply orientations, simultaneously enhancing both axial and shear load-bearing capacities.

## 4. Fatigue Tests of CNT-Reinforced Composite Specimens

### 4.1. Fatigue Test Procedure

Fatigue tests were conducted on an MTS Landmark electro-hydraulic servo fatigue testing machine using sinusoidal waveform loading under tension–tension fatigue conditions. The fatigue loading waveform is shown in Figure 10. In the figure, the vertical maximum tensile load $F_{\mathrm{Fmax}}$ is calculated according to Equation (1), while the minimum tensile load $F_{\mathrm{Fmin}}$, mean load $F_{\mathrm{Fm}}$, load amplitude $F_{\mathrm{Fa}}$, and load ratio R are calculated according to Equation (2). The loading frequency was set at 14 Hz. The test termination criteria were either complete specimen fracture or reaching the preset cycle limit of 2 × 10^6^ cycles. The fatigue specimens were prepared with the same fabrication process, geometric dimensions, and layup configuration as those used for the tensile specimens. Based on the pseudo-static tensile test results, multiple stress levels were set for each ply orientation according to their respective tensile strength values to conduct the fatigue tests. A uniform preset cycle limit was applied to all stress levels. For specimens that reached the preset cycle limit without fracture, a linear dynamic stiffness degradation model was adopted for life correction and extrapolation. A stiffness degradation exceeding 10% of the initial stiffness was used as the failure criterion. The degradation parameters were determined by least-squares fitting, and the final fatigue life was subsequently obtained.

(1) $$F_{\mathrm{Fmax}}=wtX_{t}\cdot K,$$

where w is the specimen width; t is the specimen thickness; $X_{t}$ is the tensile strength; and K is the stress coefficient. The stress coefficients for the six specimen groups are listed in Table 3. The *K* values were determined following a multi-level stress loading approach recommended in ASTM D3479/D3479M-19 [22] and ISO 13003:2003 [23]. For carbon fiber-reinforced materials, ISO 13003 recommends stress levels of 80%, 65%, 55%, and 45% of the static strength. For CNT-modified specimens, the *K* values of 0.70, 0.60, 0.50, and 0.40 were determined with reference to this standard, and were moderately reduced considering the enhanced fatigue resistance from CNT addition. For unmodified specimens, the same *K* values were first applied in pre-tests, and the stress levels were then adjusted for each ply orientation based on the observed fatigue life distribution to ensure well-distributed fatigue data on a logarithmic scale for reliable S-N curve fitting.

(2) $$\left\{\begin{array}{c} F_{\mathrm{Fm}}=\frac{F_{F\max}+F_{F\min}}{2}\\ F_{\mathrm{Fa}}=\frac{F_{F\max}-F_{F\min}}{2}\\ R=\frac{F_{F\min}}{F_{F\max}}=0.1 \end{array}\right.$$

### 4.2. Fatigue Test Results and S-N Curve Analysis

#### 4.2.1. Fatigue Life

Tension–tension fatigue tests were conducted on six groups of specimens, including those with and without CNTs, at multiple stress levels to obtain the measured fatigue lives. For specimens that reached the preset cycle limit without fracture, a linear stiffness degradation method was adopted for life extrapolation. Stiffness degradation is a well-recognized indicator of fatigue damage in composite materials, and its use is supported by ASTM D3479/D3479M-19 and ISO 13003:2003. During the tests, the cycle count N, load amplitude P, and strain amplitude δ were continuously monitored, and the dynamic stiffness K(N) at each cycle was calculated based on the secant stiffness as follows:

(3) $$K(N)=\frac{P}{\delta }$$

The stiffness–cycle count sequence was obtained. A linear degradation model was used to describe the stiffness evolution as follows:

(4) $$K(N)=K_{0}-CN$$

where $K_{0}$ is the initial dynamic stiffness and C is the degradation rate constant determined by least-squares fitting. In this study, fatigue failure was defined as dynamic stiffness degradation exceeding 10% of the initial stiffness, i.e., $K(N_{f})=0.9K_{0}$. Substituting into Equation (4) yields the extrapolated fatigue life $N_{f}$. Based on all fatigue test data, a power function model was used to directly fit the maximum stress versus fatigue life, obtaining the S-N curve equations for each specimen group, which quantitatively describe the evolution of stress level with cycle life.

#### 4.2.2. S-N Equations

The S-N data were fitted using the two-parameter Basquin equation:

(5) $$\sigma_{\max}=a{\left(N_{f}\right)}^{b}$$

where $\sigma_{\max}$ is the maximum cyclic stress, $N_{f}$ is the fatigue life, a is the fatigue strength coefficient, and b is the fatigue strength exponent. Taking the logarithm of both sides, the equation is linearized as $\log(\sigma_{\max})=A+B\log(2N_{f})$, where A=log(a) and B=b. This model form follows the recommendations of ISO 13003:2003 and ASTM E739-10 [24] for fatigue data analysis of composite materials.

The fitted S-N equation for 0° ply specimens with CNTs is:

(6) $$\log(\sigma_{\max})=-0.0898\log(2N_{f})+3.4628$$

The S-N equation for 0° ply specimens without CNTs is:

(7) $$\log(\sigma_{\max})=-0.0846\log(2N_{f})+3.3646$$

The S-N equation for ±15° ply specimens with CNTs is:

(8) $$\log(\sigma_{\max})=-0.0945\log(2N_{f})+3.2126$$

The S-N equation for ±15° ply specimens without CNTs is:

(9) $$\log(\sigma_{\max})=-0.0336\log(2N_{f})+2.7092$$

The S-N equation for ±45° ply specimens with CNTs is:

(10) $$\log(\sigma_{\max})=-0.0536\log(2N_{f})+2.2851$$

The S-N equation for ±45° ply specimens without CNTs is:

(11) $$\log(\sigma_{\max})=-0.0235\log(2N_{f})+2.1023$$

#### 4.2.3. S-N Curves

Based on the S-N curve equations obtained from the above fitting, contrast plots of the S-N curves for specimens with and without CNTs at 0°, ±15°, and ±45° ply orientations were respectively plotted, as shown in Figure 11, Figure 12 and Figure 13, which intuitively reflect the enhancement pattern of CNTs on the fatigue performance of the composites. In this study, the S-N curves are presented in double-logarithmic coordinates, with the vertical axis representing the logarithm of stress and the horizontal axis representing the logarithm of twice the fatigue life.

To assess the statistical significance of the observed differences between the S-N curves of CNT-modified and unmodified specimens, regression analysis was performed and the regression coefficients were compared. The results indicate that, due to the inherent scatter of composite fatigue data and the limited sample size, the differences do not reach the 0.05 significance level. Nevertheless, for all three ply orientations, the S-N curves of CNT-modified specimens are consistently higher than those of unmodified specimens across the entire stress range, providing evidence of the reinforcing effect of CNTs on fatigue performance with clear engineering significance. In composite fatigue research, visual separation of S-N curves, combined with systematic distribution trends of data points, is commonly used as qualitative evidence of reinforcement effects given the inherently large scatter of fatigue data. With this statistical assessment, the following sections provide a detailed comparison of the S-N curves for each ply orientation.

The fatigue failure of carbon fiber composites is primarily triggered by progressive propagation of resin matrix cracking, fiber-matrix interfacial debonding, and interlaminar delamination, ultimately leading to reduced overall load-bearing capacity. The 0° ply orientation is dominated by carbon fiber axial loading, offering the highest load-bearing efficiency and optimal fatigue performance. As shown in Figure 11, the S-N curve of specimens with CNTs is positioned entirely above and to the right of that without CNTs. At the same fatigue life, specimens with CNTs can withstand higher stress levels; at the same stress level, specimens with CNTs exhibit longer fatigue lives. In the high-stress short-life region, the separation between the two curves is greatest, indicating the most pronounced fatigue performance enhancement by CNTs. As cycle life increases, the two curves gradually converge, with the performance difference progressively diminishing. Regarding curve slope, the S-N curves of specimens with CNTs exhibit a larger absolute slope, indicating greater sensitivity of fatigue performance to changes in stress level. CNTs can more effectively suppress rapid crack propagation under high-stress conditions, thereby slowing fatigue failure. For the 0° ply orientation, where specimens are primarily under axial loading along the carbon fibers, fatigue damage is mainly concentrated at the fiber-matrix interface. CNTs can effectively enhance interfacial bonding strength, improve axial load transfer efficiency, and suppress interfacial debonding and fiber pull out, thereby slowing fatigue crack initiation and propagation.

The ±15° ply orientation involves combined axial and shear loading, with the interface and matrix bearing more of the load. As shown in Figure 12, the S-N curves of the specimens with CNTs are also steadily located to the upper right of those without CNTs, exhibiting a fatigue performance enhancement pattern consistent with that of the 0° ply orientation. In the high-stress short-life region, the two sets of curves are clearly separated. CNTs effectively suppress interfacial debonding and matrix cracking under shear loading by strengthening the matrix and improving interfacial bonding, resulting in a pronounced improvement in fatigue life. In the low-stress long-life region, the curves gradually converge, and the difference in fatigue performance diminishes. Compared with the 0° ply orientation, the slope of the S-N curve for specimens with CNTs under the ±15° ply orientation is larger, indicating a more significant regulatory effect of CNTs on fatigue performance under combined loading conditions. For the ±15° ply orientation, where specimens are subjected to combined axial and shear loading, the interface and matrix simultaneously bear cyclic stresses. CNTs can simultaneously enhance the toughness of the resin matrix and the interfacial bonding, dissipating energy through mechanisms such as crack deflection and bridging, thereby mitigating internal damage accumulation under combined loading.

The ±45° ply orientation is dominated by in-plane shear deformation, with the resin matrix and fiber-matrix interface being the key factors determining fatigue performance. As shown in Figure 13, in the high-stress short-life region, the S-N curves of specimens with CNTs are positioned significantly above those without CNTs, demonstrating a clear improvement in fatigue performance. The strengthening effect of CNTs on the matrix and interface plays a dominant role, effectively suppressing the rapid propagation of fatigue damage under shear deformation. In the low-stress long-life region, the two curves exhibit localized crossover. This phenomenon is mainly attributed to the inherent experimental scatter of composite fatigue life: the fatigue performance of carbon fiber/epoxy resin composites is influenced by various microscopic factors such as resin impregnation, interfacial bonding quality, and internal micro-voids. Under low-stress conditions, damage accumulates slowly, and the influence of minor initial defects on fatigue life is amplified, leading to higher data scatter. This results in partial overlap of the fatigue life data between the two groups, which manifests as crossover in the curves. This is a normal phenomenon in fatigue testing of composites and does not affect the overall conclusion that CNTs enhance fatigue resistance. For the ±45° ply orientation, where specimens are dominated by in-plane shear deformation, the matrix and interface become the key factors controlling fatigue performance. CNTs significantly improve matrix strength and interfacial bonding, suppressing matrix cracking and interfacial debonding under shear loading, thereby enabling the material to maintain a relatively high fatigue resistance even under shear-dominated conditions.

Collectively, Figure 11, Figure 12 and Figure 13 demonstrate that across all three ply orientations, the S-N curves of specimens with CNTs are consistently higher than those without CNTs, indicating comprehensive fatigue performance enhancement. The absolute slope values are also larger than those of specimens without CNTs, reflecting greater fatigue load bearing stability. As the ply orientation increases from 0° to ±45°, the S-N curves of specimens without CNTs shift continuously downward, with rapid degradation in fatigue performance, while the downward shift in the curves for specimens with CNTs is considerably smaller. This indicates that CNTs can effectively compensate for the performance loss induced by shear loading and broaden the service condition range of the composites.

As stress level decreases and cycle life extends, the S-N curves of the two groups gradually converge with diminishing performance differences. This is because under high stress, crack initiation and propagation rates are fast, and CNTs can rapidly dissipate energy through crack bridging, deflection, and pull-out mechanisms, suppressing matrix cracking and interfacial debonding, with the reinforcing effects fully manifested. Under low-stress long-life conditions, fatigue life scatter increases while internal damage accumulates slowly, weakening the instantaneous toughening benefits of CNTs, causing the curves to converge. Overall, through the synergistic effects of matrix strengthening, interface optimization, and multi-scale energy dissipation, CNTs can effectively suppress fatigue damage initiation and propagation, slow stiffness degradation, and comprehensively enhance both the static mechanical properties and fatigue life of carbon fiber/epoxy resin composites.

## 5. Conclusions

In this paper, pseudo-static tensile and multi-stress-level tension–tension fatigue tests on CFRP laminate specimens with ply angles of 0°, ±15°, and ±45°, with and without CNTs incorporated into the epoxy matrix, have been presented. The following conclusions are drawn:

In terms of static mechanical properties, adding CNTs of 3% mass fraction to the matrix increased greatly the tensile strength and elastic modulus of the composites. The tensile strength and elastic modulus of CNT-modified specimens were considerably higher than those of unmodified counterparts. The measured stress–strain curves revealed that CNTs could effectively improve matrix toughness and the uniformity of load transfer, and mitigate the adverse impacts of internal defects and stress concentration on material failure behavior. Static bearing performance varied significantly with ply orientation. Laminates with a 0° ply angle took advantage of the axial bearing capacity of carbon fibers and achieved prominent improvements in tensile properties after CNT modification. Specimens with ±15° ply angles endured combined axial and shear loads and exhibited steady growth in all mechanical parameters. For ±45° laminates dominated by shear deformation, CNTs reinforced the resin matrix and strengthened fiber-matrix interfacial bonding simultaneously, resulting in improved pseudo-static tensile strength and structural stiffness for all three ply orientations.

Regarding fatigue performance, the S-N curves of specimens with CNTs featured larger absolute slopes and steeper trends then those of specimens without CNTs, for ply angles ranging from 0° to ±15° and ±45°. The 0° laminates, loaded mainly along the fiber axial direction, obtained remarkable fatigue resistance enhancement after modification. The ±15° specimens under coupled axial-shear loads showed synchronous improvements in fatigue durability. For shear-dominated ±45° laminates, local crossing of S-N curves only emerged in the low-stress long-life region due to experimental scattering, which did not alter the overall improvement trend of CNT reinforcement. In general, CNT-modified specimens bore higher stress levels under identical fatigue cycles and sustained far longer fatigue life under equivalent applied stress. The reinforcing effect of CNTs on fatigue performance was more pronounced under high-stress short-cycle loading; the performance gap between modified and blank specimens gradually narrowed as applied stress decreased and fatigue life extended. CNTs effectively slowed the degradation of structural stiffness and extended the service life of composites under cyclic loading, along with improved operational stability.

## Figures and Tables

**Figure 1:**
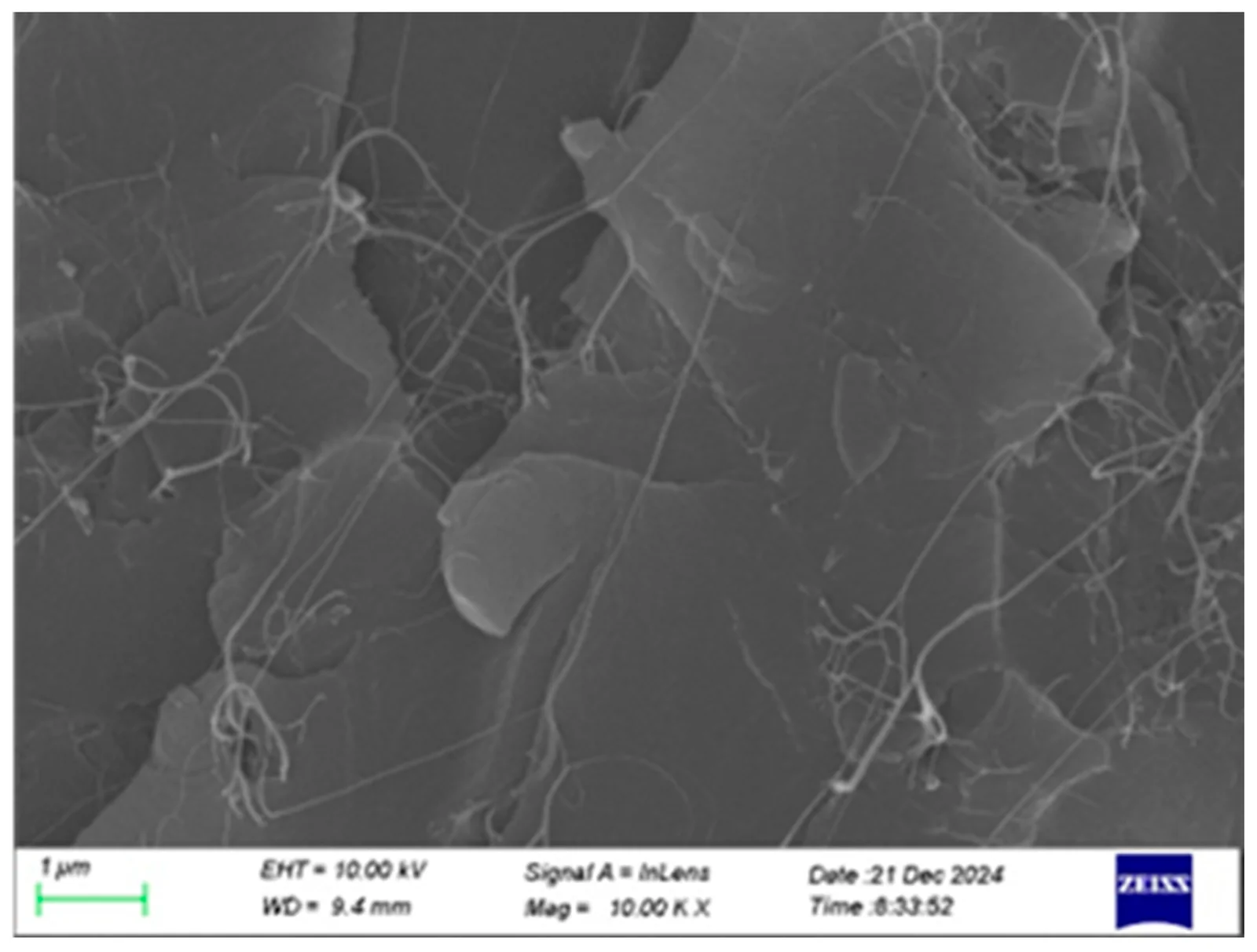
SEM image of the fracture surface of 3 wt% SWCNT/epoxy casting.

**Figure 2:**
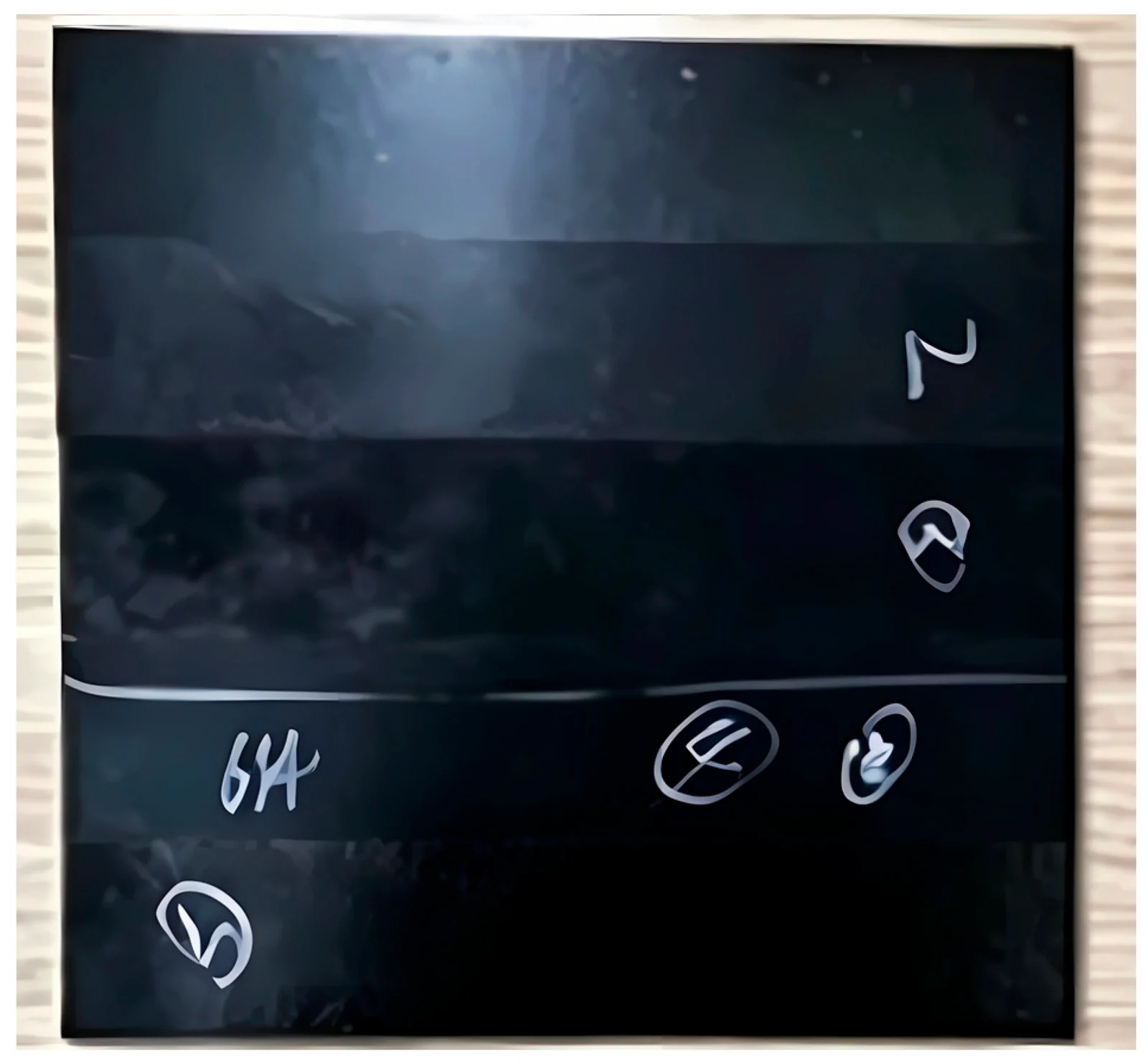
Photograph of 5 standard laminate specimens. The handwritten marks on specimens are internal labels used only to distinguish individual samples during experiments.

**Figure 3:**
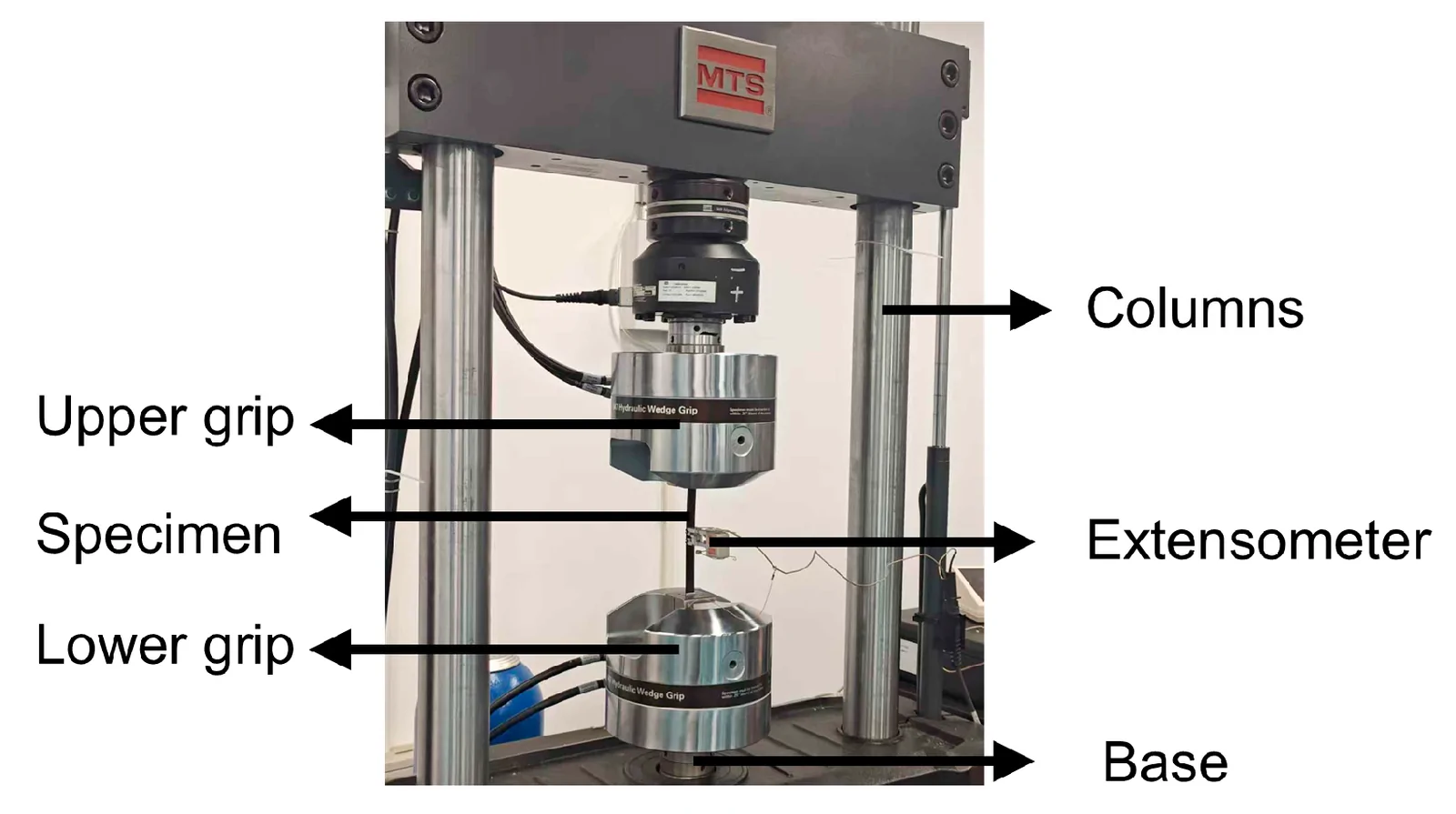
Photograph of tensile test setup.

**Figure 4:**
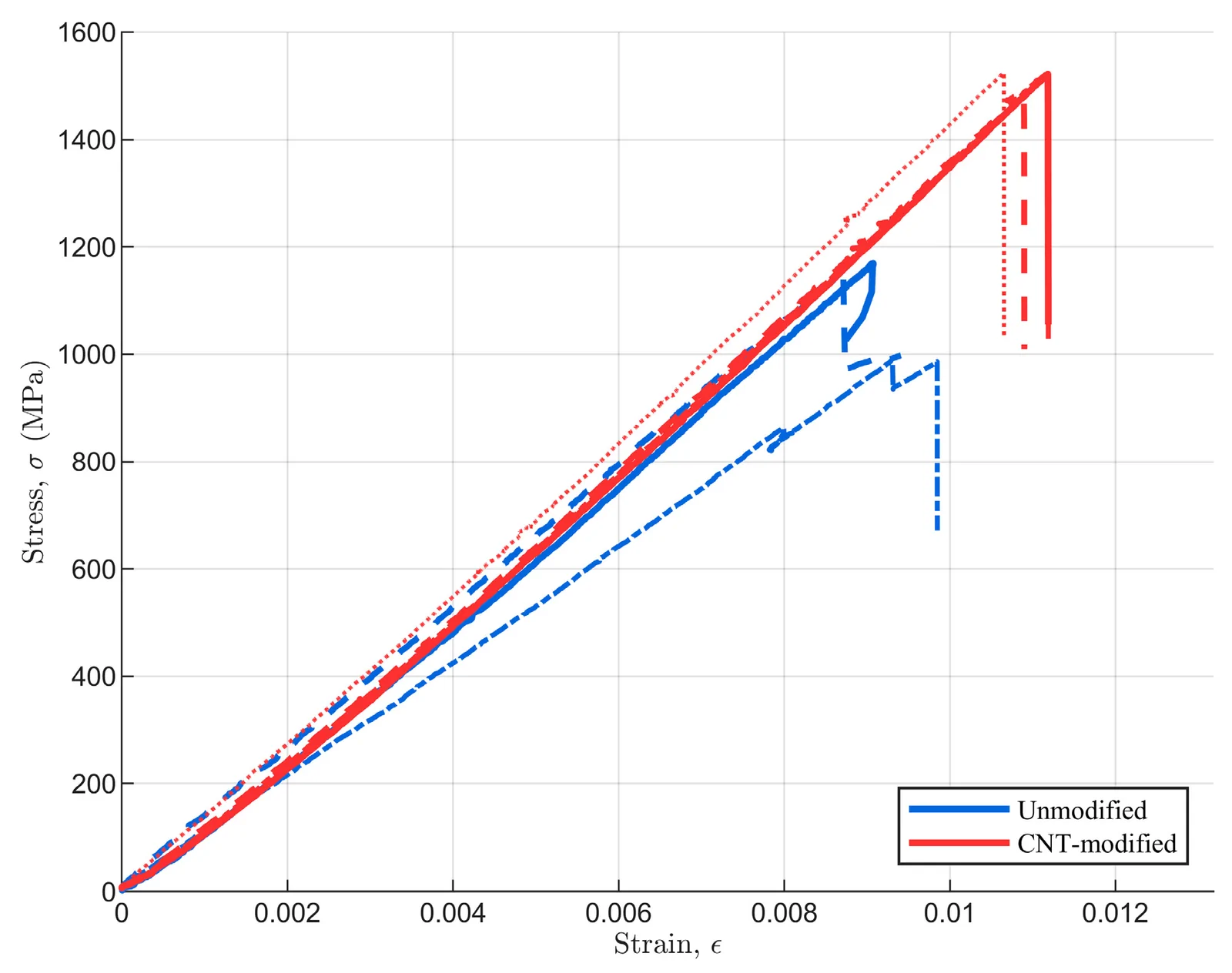
Stress–strain curves of 0° ply specimens with and without CNTs. Red and blue curves represent CNT-modified and unmodified specimens, respectively. Solid, dashed, dash-dotted, and dotted lines indicate individual replicate specimens within each group.

**Figure 5:**
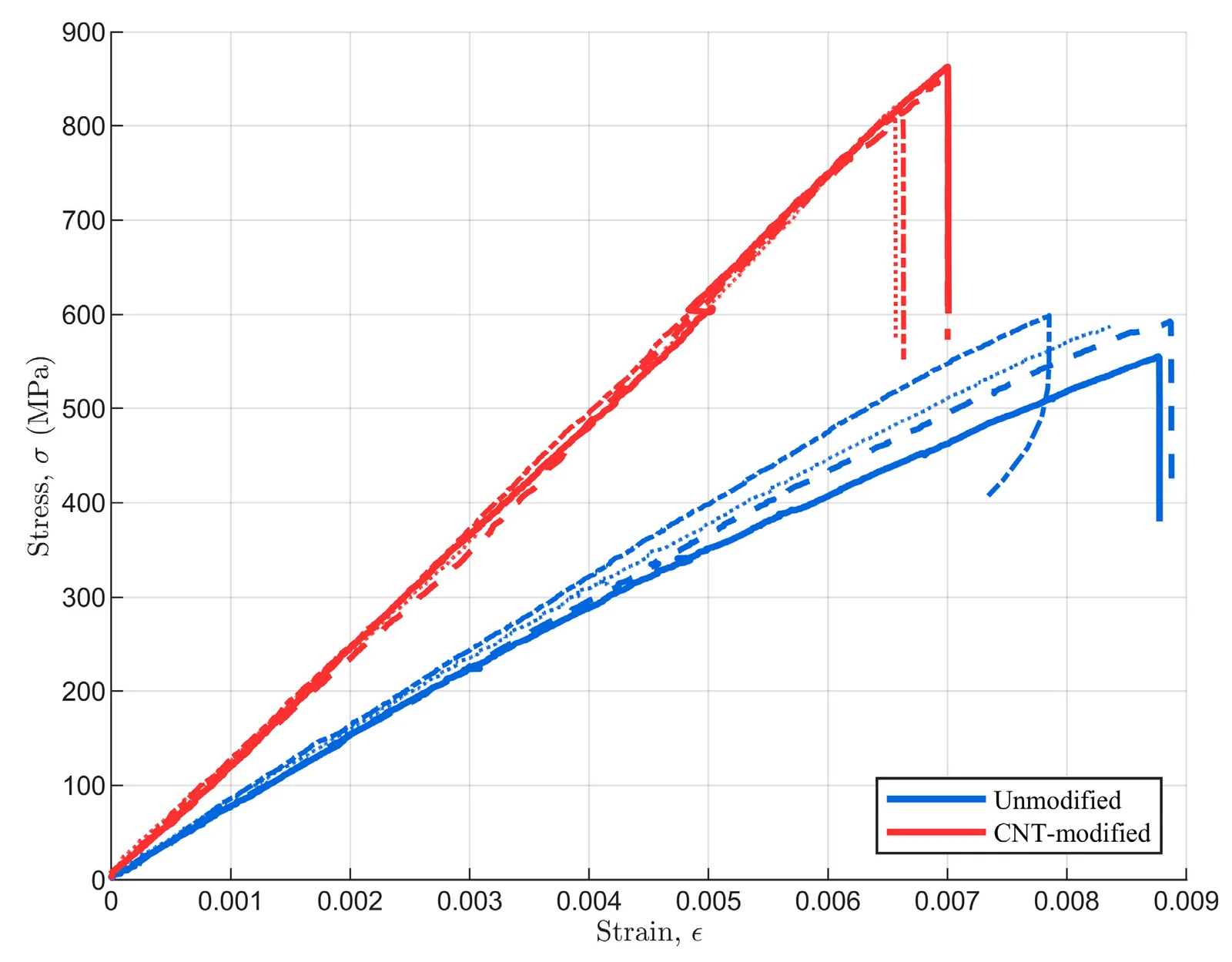
Stress–strain curves of ±15° ply specimens with and without CNTs. Red and blue curves represent CNT-modified and unmodified specimens, respectively. Solid, dashed, dash-dotted, and dotted lines indicate individual replicate specimens within each group.

**Figure 6:**
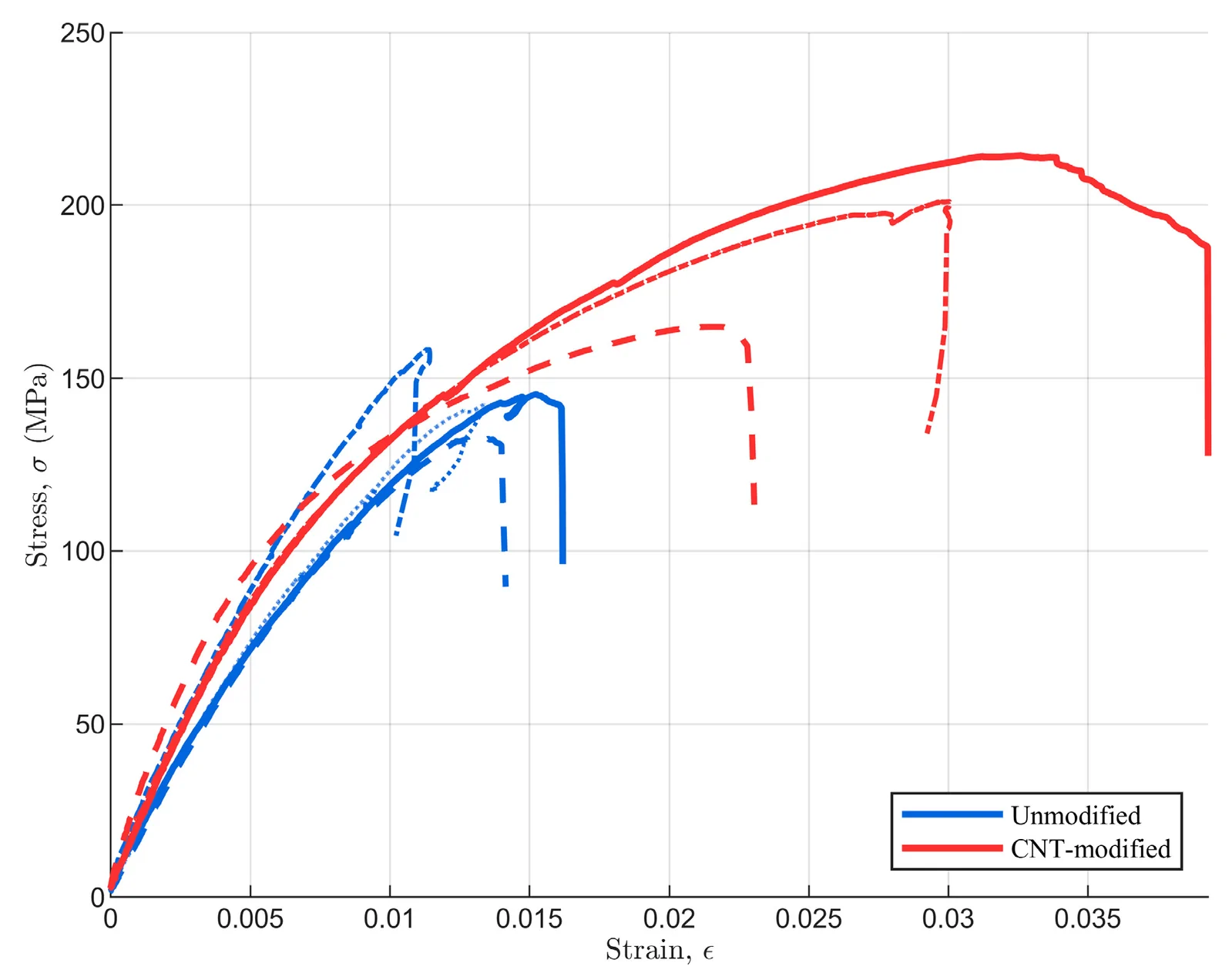
Stress–strain curves of ±45° ply specimens with and without CNTs. Red and blue curves represent CNT-modified and unmodified specimens, respectively. Solid, dashed, dash-dotted, and dotted lines indicate individual replicate specimens within each group.

**Figure 7:**
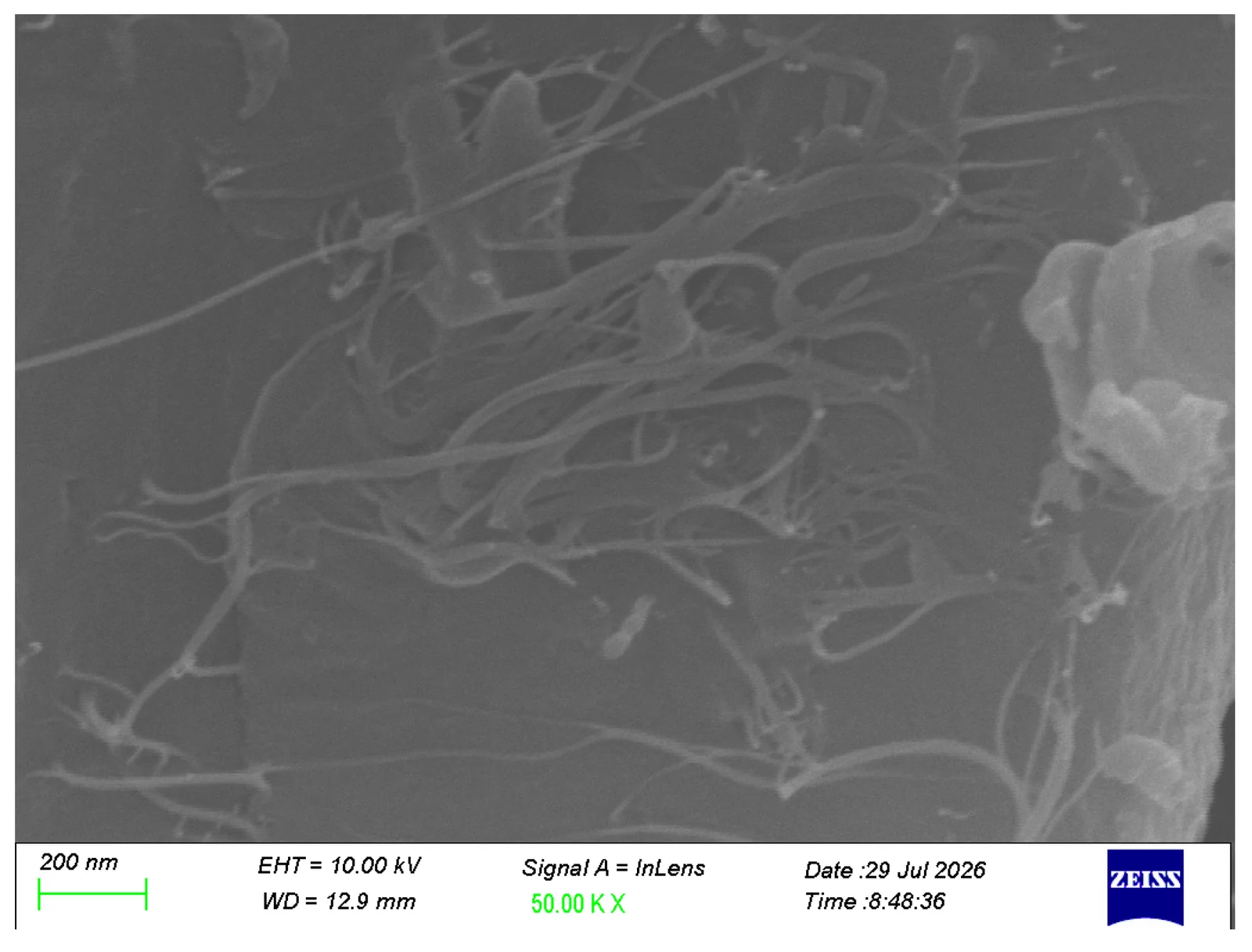
SEM image of the fracture surface of CNT-modified tensile specimen with 0° ply.

**Figure 8:**
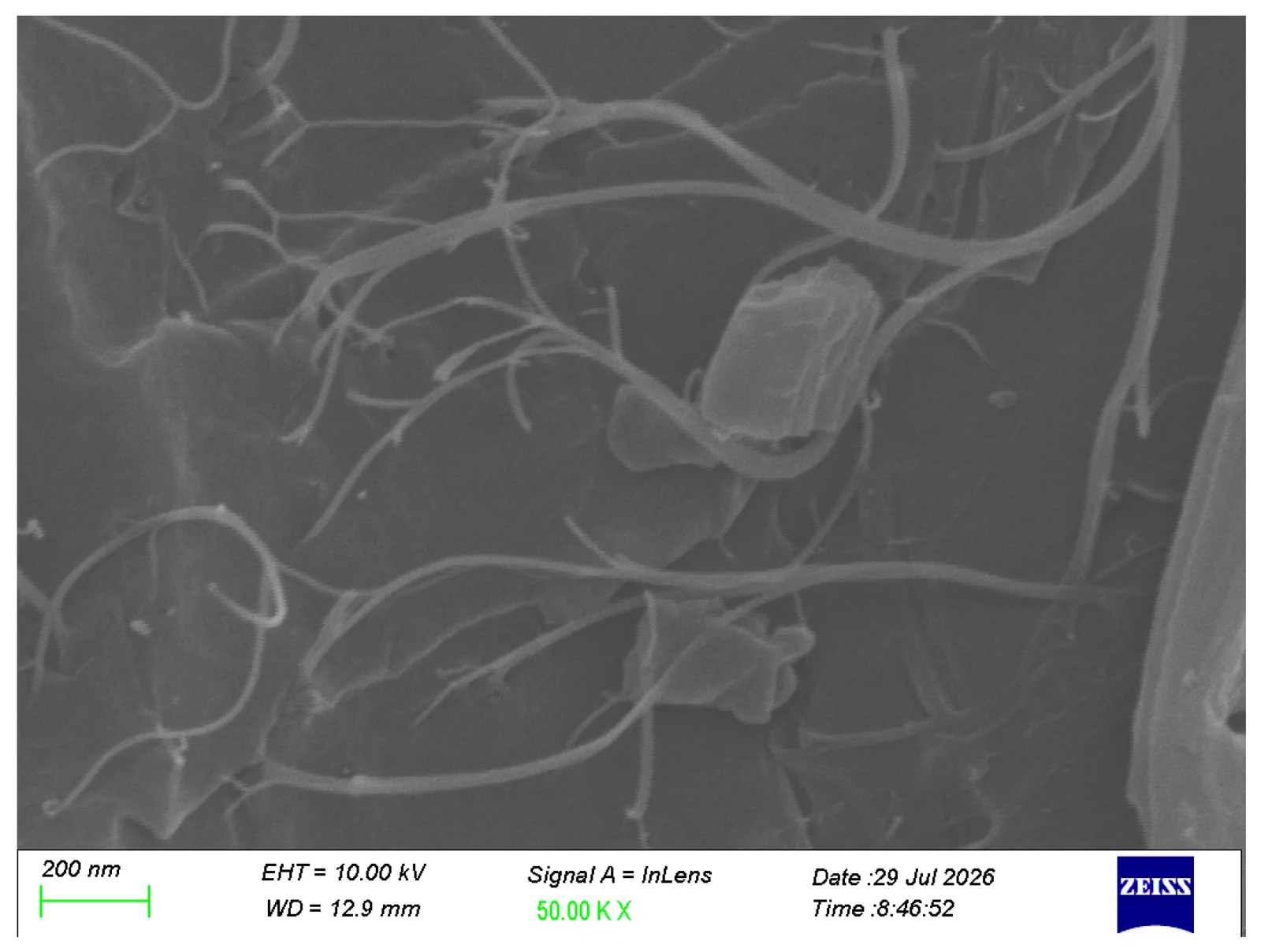
SEM image of the fracture surface of CNT-modified tensile specimen with ±15° ply.

**Figure 9:**
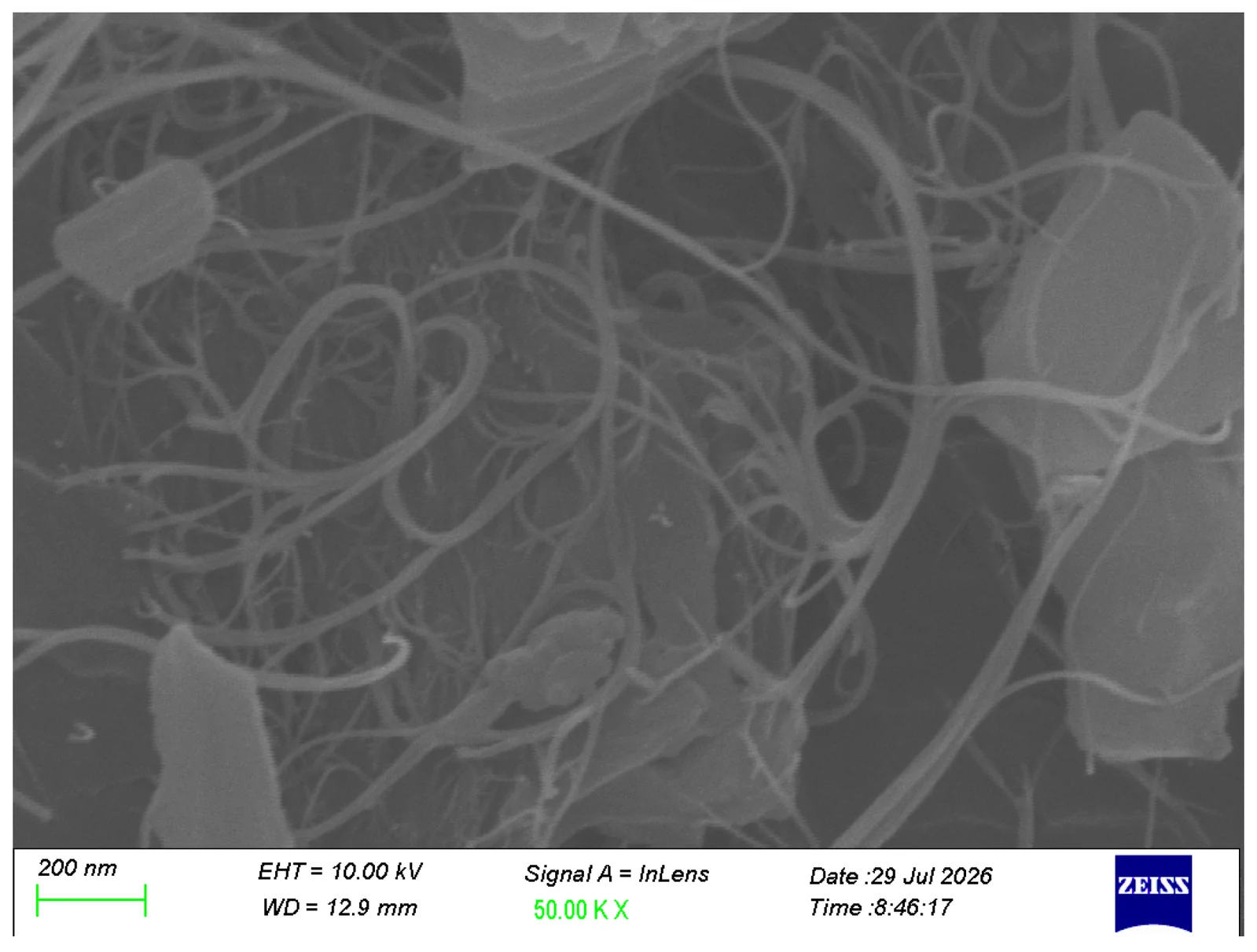
SEM image of the fracture surface of CNT-modified tensile specimen with ±45° ply.

**Figure 10:**
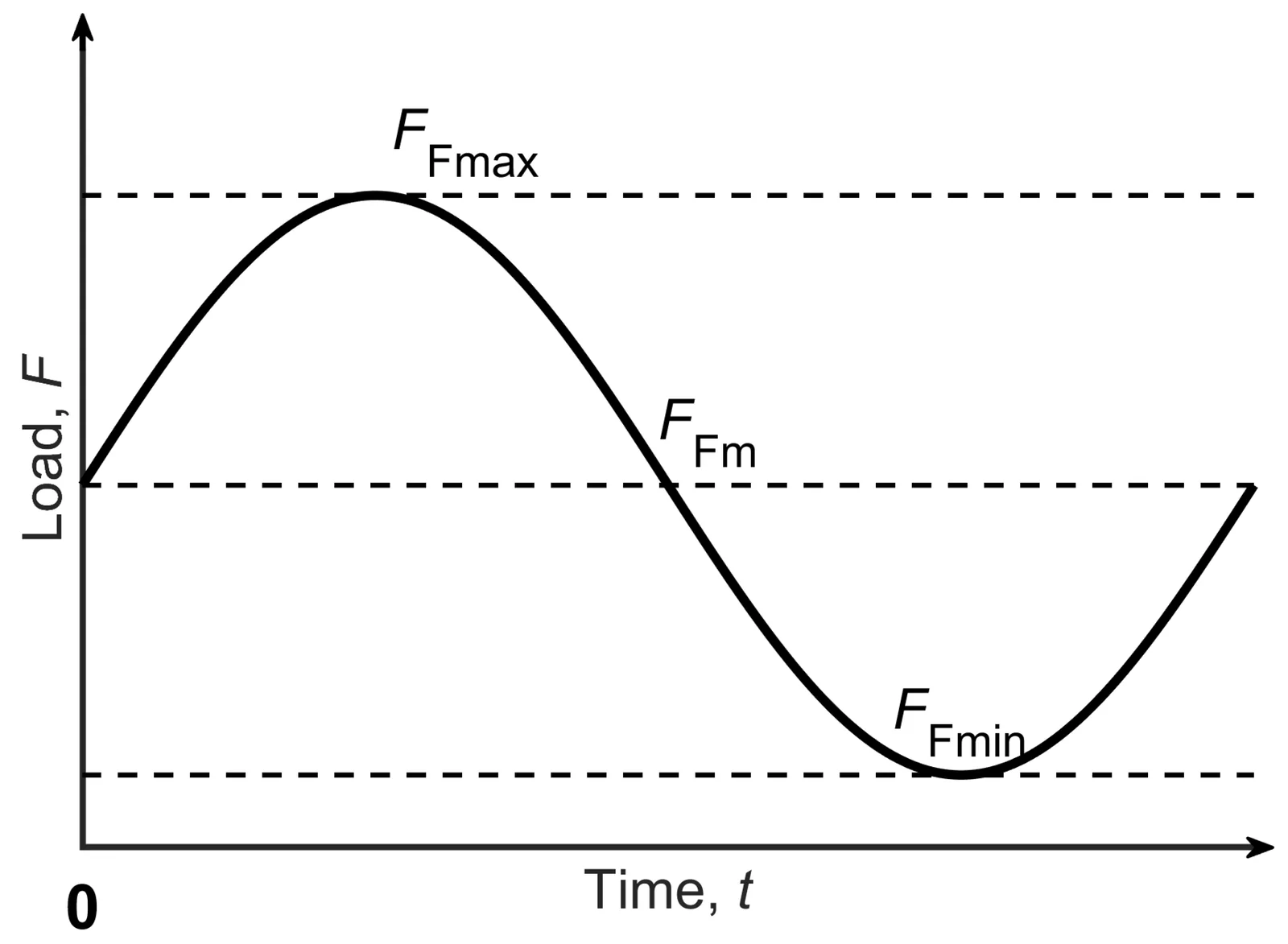
Fatigue load waveform, where the dotted lines denote the maximum load $F_{F\max}$, mean load $F_{\mathrm{Fm}}$, and minimum load $F_{F\min}$.

**Figure 11:**
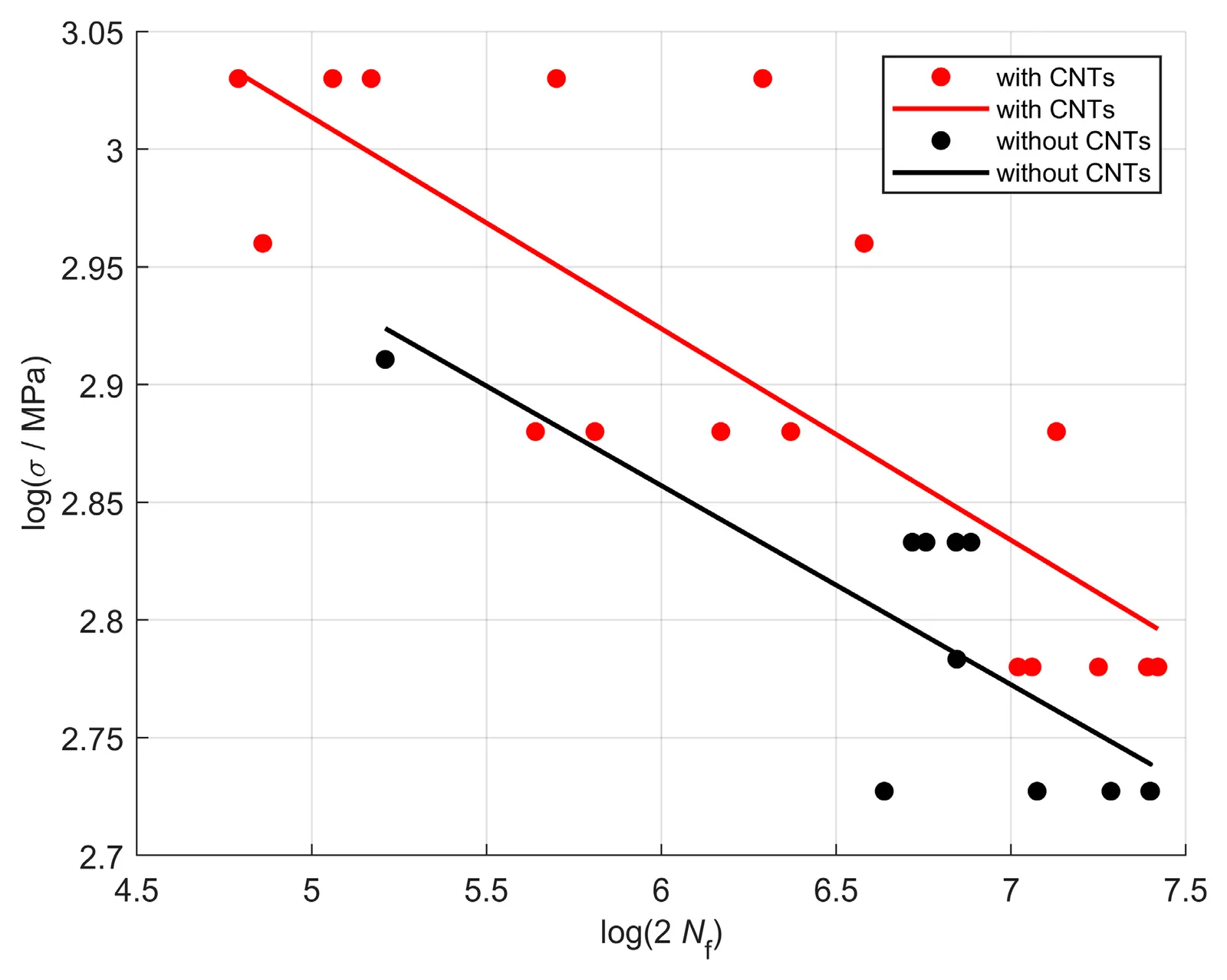
Comparison of S-N curves for 0° ply specimens with and without CNTs.

**Figure 12:**
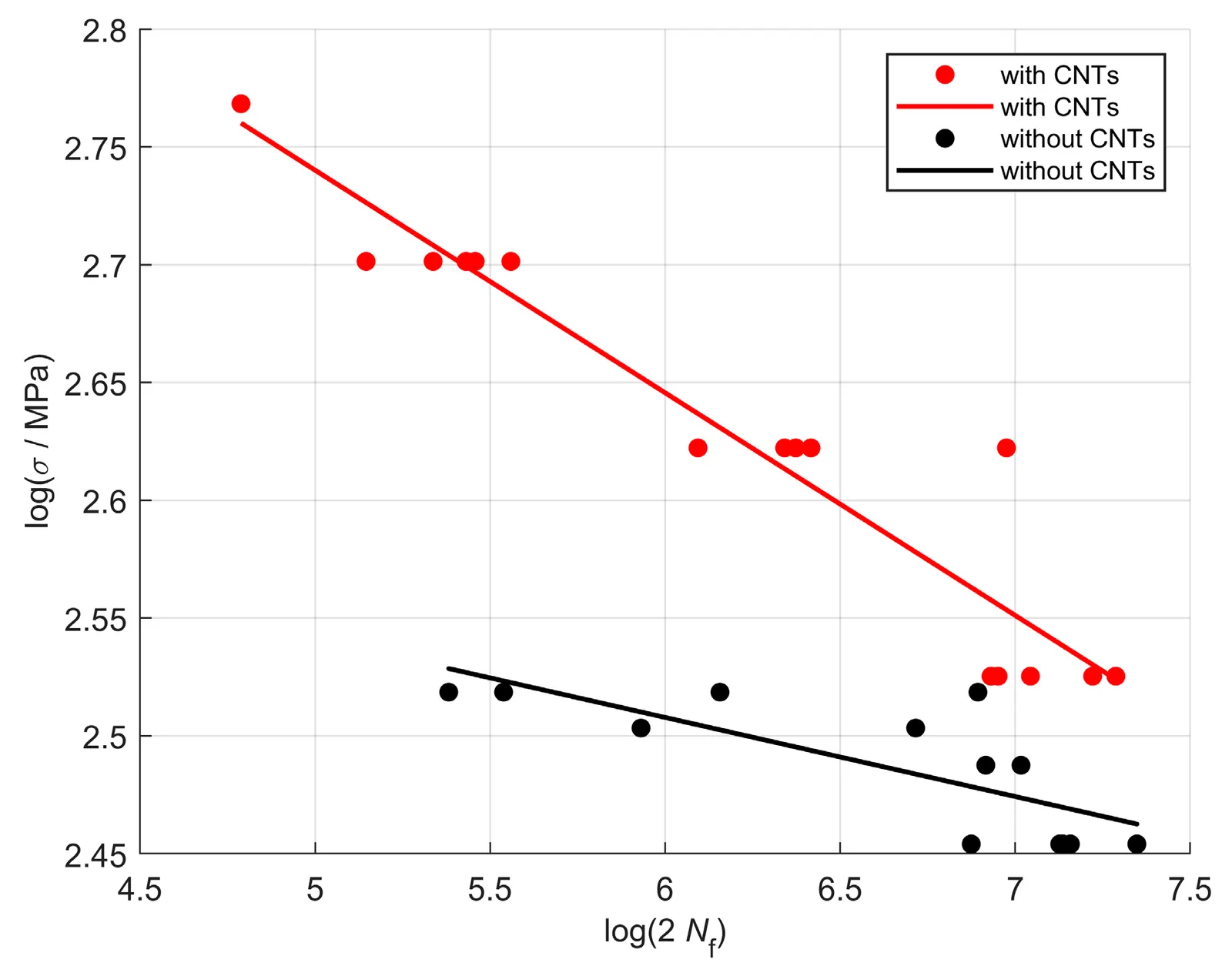
Comparison of S-N curves for ±15° ply specimens with and without CNTs.

**Figure 13:**
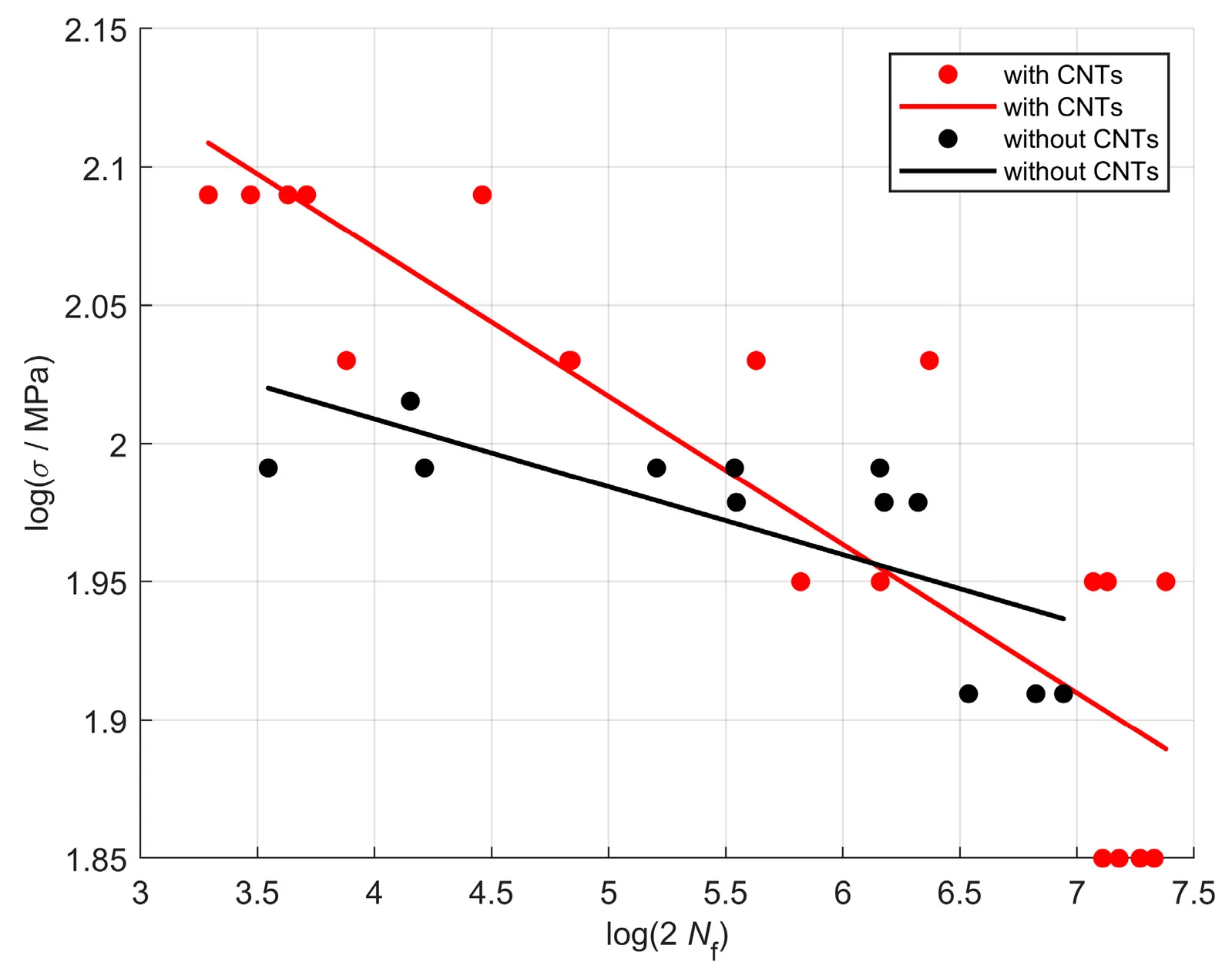
Comparison of S-N curves for ±45° ply specimens with and without CNTs.

**Table 1:** Tensile properties of specimens with and without CNTs at different ply angles.

Ply Angle	Specimen Type	*n* ^1^	Tensile Strength (MPa) ^2^	Elastic Modulus (GPa)
0°	Without CNTs	3	1104.67 ± 100.4	121.00 ± 15.1
0°	With CNTs	4	1515.50 ± 17.0	127.00 ± 6.5
±15°	Without CNTs	4	585.50 ± 19.8	73.30 ± 4.9
±15°	With CNTs	4	838.00 ± 23.5	118.78 ± 5.5
±45°	Without CNTs	4	144.25 ± 10.7	14.08 ± 1.3
±45°	With CNTs	3	193.33 ± 25.4	19.07 ± 1.4

^1^ *n*: number of valid specimens. ^2^ Values are presented as mean ± standard deviation.

**Table 2:** Improvement in tensile properties induced by CNT addition and statistical significance.

Ply Angle	Property	Improvement (%)	95% CI (%) ^1^	*p* ^2^
0°	Tensile strength	37	[20.3, 54.1]	<0.05
0°	Elastic modulus	5	[−19.5, 29.4]	<0.05
±15°	Tensile strength	43	[36.7, 49.6]	<0.01
±15°	Elastic modulus	62	[49.7, 74.4]	<0.01
±45°	Tensile strength	34	[−0.3, 68.4]	<0.05
±45°	Elastic modulus	35	[11.9, 59.0]	<0.05

^1^ 95% CI: 95% confidence interval for the percentage improvement. ^2^ *p* values are from two-tailed Student’s *t*-tests comparing CNT-modified and unmodified specimens.

**Table 3:** Stress coefficients for six ply orientations.

Stress Level	0° with CNTs	0° Without CNTs	±15° with CNTs	±15° Without CNTs	±45° with CNTs	±45° Without CNTs
1	0.70	0.88	0.70	0.58	0.70	0.74
2	0.60	0.74	0.60	0.56	0.60	0.70
3	0.50	0.66	0.50	0.54	0.50	0.68
4	0.40	0.58	0.40	0.50	0.40	0.66
5	*	*	*	*	*	0.58

* Data not available.

## Data Availability

The raw data supporting the conclusions of this article will be made available by the authors on request.
